# Effects of Virtual Reality Use on Children with Cerebral Palsy and Its Applications in Health: A Systematic Review

**DOI:** 10.3390/healthcare13202571

**Published:** 2025-10-13

**Authors:** Angie Estefania Mesa-Burbano, María Alejandra Fernández-Polo, John Steven Hurtado-Sánchez, Silvia Patricia Betancur-Bedoya, Diana Maritza Quiguanas-López, Leidy Tatiana Ordoñez-Mora

**Affiliations:** 1 Physiotherapy Program, Faculty of Health Sciences, Fundación Universitaria María Cano, Medellín 050012, Colombia; angieestefaniamesaburbano@fumc.edu.co (A.E.M.-B.); mariaalejandrafernandezpolo@fumc.edu.co (M.A.F.-P.); silviapatriciabetancurbedoya@fumc.edu.co (S.P.B.-B.); 2 Physiotherapy Program, Health Faculty, Universidad de San Buenaventura, Cartagena 130001, Colombia; John.hurtado@usbctg.edu.co; 3 Physical Activity and Sport Program, Education Faculty, Corporación Universitaria Adventista de Colombia, Medellín 050032, Colombia; 4 Physiotherapy Program, Health Faculty, Universidad Santiago de Cali, Cali 760035, Colombia; diana.quiguanas00@usc.edu.co

**Keywords:** cerebral palsy, virtual reality, rehabilitation, physical therapy, neurology

## Abstract

**Background/Objectives**: This study evaluated the effects of virtual reality (VR) on functionality, quality of life, and motivation in children with cerebral palsy (CP). **Methods**: The systematic review was registered in PROSPERO (CRD42022321170) and conducted using the keywords physical therapy OR physiotherapy AND “Virtual Reality”. Studies were screened based on title, abstract, and full-text review. The PEDro scale was used to assess methodological quality, and the GRADE system was applied to determine the level of certainty of the evidence. **Results**: A total of 10 studies showed improvements in balance, 6 in gross motor function, and 8 in upper limb coordination. Additional gains were found in daily functioning (6), gait (4), motivation (3), and spasticity (1–2). Overall, virtual reality enhanced motor abilities and engagement compared with conventional therapy, underscoring its value as a playful and motivating tool in rehabilitation. All outcome measures showed positive changes, particularly in functionality and quality of life. The primary outcomes with the most favorable responses to intervention were gross motor function and balance, followed by motivation and adherence. However, the generalities of the findings are limited due to variabilities in outcome reporting and measurement tools. **Conclusions**: The findings indicated clinical improvements in key outcome measures following VR interventions. Nonetheless, there were significant variabilities in the evaluation instruments used across studies. Despite this, the clinical evidence supported the integration of VR into neurorehabilitation processes for children with CP.

## 1. Introduction

Cerebral palsy (CP) is the most common cause of motor disability in children. It is defined as a non-progressive disorder of movement and posture, and it also encompasses non-motor clinical manifestations that significantly affect quality of life, including cognitive impairments, epilepsy, sensory deficits, language and sleep disorders, as well as digestive, respiratory, and musculoskeletal problems [1]. The prevalence of CP is estimated at approximately 3 per 1000 live births [2]. This makes CP a public health concern due to its long-term neuromusculoskeletal implications, particularly those affecting motor development and limiting the ability to perform activities of daily living [3].

The impact of varying levels of disability on quality of life in individuals with CP has motivated the exploration of approaches that are both feasible to implement and supported by scientific evidence. Such approaches consider barriers and facilitators related to the use of devices, technologies, services, systems, and policies that may positively influence independence and social inclusion. Among these, rehabilitation technologies—particularly virtual reality (VR)—have gained prominence. VR refers to interactive simulations created using hardware and software, allowing users to engage with virtual environments or systems [4]. Increasingly, video games have been incorporated into rehabilitation programs [3], enabling individuals to participate in simulated movement-based tasks that foster engagement. This approach supports neuroplasticity, personal motivation, and confidence in motor skills, facilitated by neuronal reorganization processes [5,6].

VR-based rehabilitation leverages neurophysiological mechanisms that enhance neuroplasticity by promoting cortical and subcortical reorganization through repetitive, multisensory practice. Such interventions strengthen synaptic connections, activate motor and cerebellar regions, improve postural stability and coordination through multisensory recalibration, and stimulate dopaminergic pathways that boost motivation and adherence to therapy [7].

The persistent functional impairments experienced by children and adolescents with CP have driven interest in therapeutic strategies grounded in scientific evidence. These strategies aim not only to address functional limitations but also to integrate technologies that enhance independence and social participation. Within this framework, VR has been adopted in rehabilitation settings, providing immersive scenarios where children and adolescents can practice functional movement patterns in simulated real-life contexts [4]. By using video games, VR promotes participation [3], enhances motor learning, and facilitates the acquisition of motor and sensory skills. Furthermore, VR interventions improve motivation, self-confidence, and body awareness through neuroplasticity processes that reinforce motor learning [5,6].

VR has therefore been applied in therapeutic interventions for individuals with CP, offering immersive and interactive experiences that expand treatment possibilities. Virtual environments allow real-world tasks to be transferred into controlled clinical contexts, supporting both motor learning and professional assessment [5]. These conditions promote the development of comprehensive rehabilitation tools that enhance adherence and participation, addressing body functions and structures, activity and participation, and environmental factors [8]. Nevertheless, no research has yet detailed the characteristics of standardized VR-based physiotherapy protocols specifically designed for individuals with CP. From a health perspective, physiotherapeutic approaches employing VR require further systematization, considering differential needs and the life course [9].

Systematic reviews suggest that VR interventions in CP rehabilitation enhance neuroplasticity and sensorimotor integration, improve postural control and balance [10], apply motor learning principles such as repetition and feedback [11], and facilitate fine motor control through cortical reorganization [12]. Recent evidence also supports VR-based telerehabilitation, demonstrating significant improvements in hand function, gross motor skills, and walking capacity in home-based settings [13].

Therefore, the objective of this study was to evaluate the effects of VR interventions in children with cerebral palsy, compared with alternative or no interventions, on outcomes related to functionality, quality of life, and motivation.

## 2. Materials and Methods

A systematic review was conducted under the recommendations of the Cochrane Collaboration and the PRISMA declaration [14]. The protocol was registered in PROSPERO (CRD42022321170).

### 2.1. Inclusion Criteria

The following research question was established:

- Population: children between 4 and 18 years of age with a diagnosis of cerebral palsy at any level of Gross Motor Function Classification System (GMFCS) who could follow instructions and who had no severe visual or auditory limitations.
- Interventions: VR, excluding treadmill, lokomat, and equine therapy simulator equipment.
- Comparison of interventions: this review included studies that had applied some type of VR versus conventional therapy, another intervention, or no intervention but without combining interventions in each group.
- Primary outcome measures included: functionality (motor function, balance, gait, and manual function). Activities and participation, motivation and/or satisfaction.

We included randomized controlled trials (RCTs). Both parallel-group and crossover RCTs were eligible, as well as pilot RCTs, provided they reported relevant outcome measures.

### 2.2. Exclusion Criteria

Studies with no differentiation of the neurological alteration and those lacking clarity regarding the main diagnosis were excluded.

Publications that did not detail the protocol or instruments and/or scales used for the VR approach were omitted.

### 2.3. Search Methods

Articles in English and Spanish that were published during the period 2012–2024 and involved bibliometric analysis in preliminary searches performed in SCOPUS were included: (cerebral AND palsy AND virtual AND reality AND rehabilitation), where this was established as the range with the highest tendency.

The databases and bibliographic engines PeDro, LILACS, Springer Link, Cochrane, Google Scholar, and EBSCO were used. The search strategy was based on the following terms “cerebral palsy” AND (“physical therapy” OR “physiotherapy”) AND “Virtual Reality” (See Appendix A). Keywords in Mesh terms (English language), such as Cerebral Palsy, Physiotherapy, Virtual Reality, Technology, and Rehabilitation, were also considered. All searches were performed online and managed using Endnote.

### 2.4. Study Selection

The calibration process was the first step in the selection of studies. Two researchers initiated the filtering processes blindly and independently after searching the different databases. Each one produced a list of studies after analyzing the title and abstract of each article. The inclusion of the article was decided when there was concordance between the reviewers, and should there be any discrepancies regarding the answer, a third evaluator, blinded to the answers given by each one, decided on its inclusion. The eligibility criteria were applied to the analysis of the full text in the final selection. Any disagreement between authors regarding eligibility, quality, and data retrieved from the studies was solved by consensus.

### 2.5. Data Extraction

Data extraction was performed independently using the Rayyan tool, which included first author and year, research design, scales used, and main outcomes. Based on these outcomes, the measure corresponding to the mean and standard deviation per reported outcome was documented, and all investigators confirmed that the information was the corresponding one.

### 2.6. Assessment of Risk of Bias and Methodological Quality

For the assessment of the methodological quality and risk of bias, PeDro scale [15] was used to assess randomization, allocation concealment, participant masking, therapist masking, assessor masking, outcome data in at least 85% of participants for at least one primary outcome, intention-to-treat analysis, statistical comparisons between groups, and their outcomes.

### 2.7. Certainty Assessment

The GRADE tool [16] was used to assess the level of certainty and according to this approach, the factors that can decrease the evidence may include study limitations, inconsistency of results, indirect evidence, imprecision, and publication bias. Based on the above, the analysis of the articles included considered the following categories:

High: There is high confidence that the true effect is close to the effect estimate.

Moderate: There is moderate confidence in the estimate of the effect; the true effect is likely to be close to the estimate of the effect, but there is a possibility that it is substantially different.

Low: Confidence in the estimate of the effect is limited; the true effect may be substantially different from the estimate of effect.

Very low: There is very low confidence in the estimate of effect; the true effect is likely to be substantially different from the estimate of effect.

## 3. Results

### 3.1. Identification of Studies

**Figure 1:**
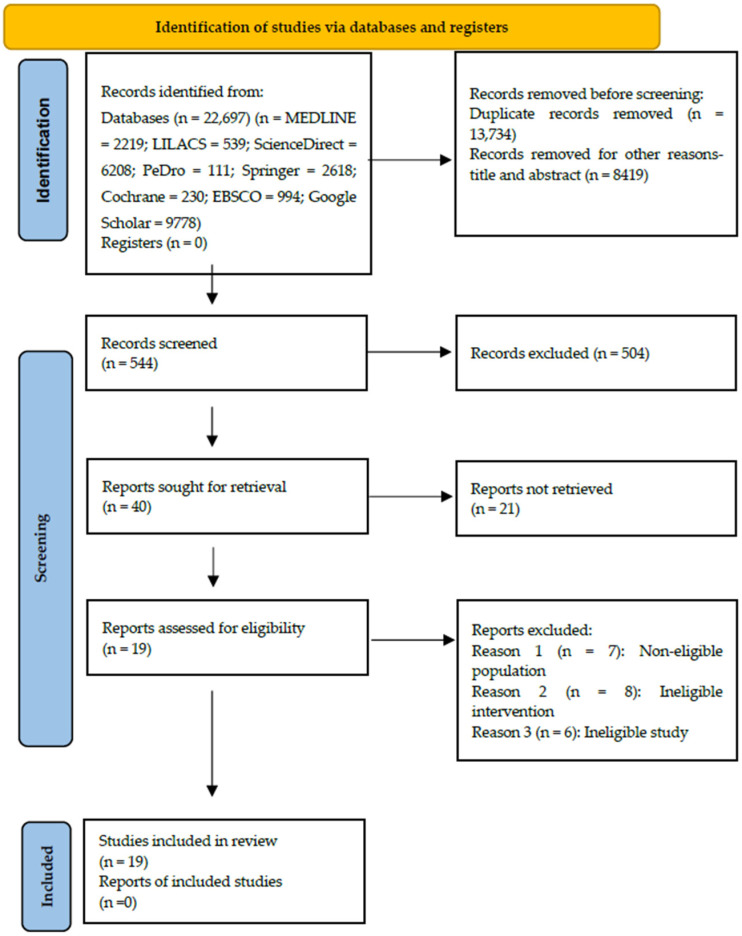
PRISMA flowchart of studies included and excluded in the systematic review. PRISMA 2020 flow diagram illustrating the study selection process [14] (www.prisma-statement.org accessed on 21 September 2025).

A total of 22,697 articles were initially selected after searching the Medline, Science Direct, PeDro, Lilacs, Springer link, Cochrane, Google Scholar, and EBSCO databases. After eliminating the duplicates, 8963 research papers were identified, of which 544 full-text articles were checked for eligibility (Figure 1). After applying the inclusion criteria, 40 articles were identified. Subsequently, after carefully reading each article and analyzing the exclusion criteria previously described, 19 [17,18,19,20,21,22,23,24,25,26,27,28,29,30,31,32,33,34,35] investigations were finally included for the present systematic review.

### 3.2. Characteristics of the Studies Included

In the 19 research studies analyzed [17,18,19,20,21,22,23,24,25,26,27,28,29,30,31,32,33,34,35], a total of 704 children with CP were included, whose age ranged between 4 and 20 years of age (one investigation included people up to 20 years of age according to the New Zealand underage normative framework). The studies were conducted in countries such as Taiwan, Egypt, Republic of Korea, Saudi Arabia, Bangalore, Belgium, India, Turkey, Iran, and Sweden. All studies included were randomized control group clinical trials (Table 1).

### 3.3. Characteristics of the Population

From the studies reviewed, 10 research studies included a population sample of children with spastic hemiplegia CP [17,18,19,20,21,22,23,24,25,26,32,34,35], followed by children with spastic diplegia CP [22,25,27,28,35], quadriplegia CP [23,27,28], monoplegia [35], dyskinetic CP [22,23] and ataxic [33]. One investigation included triplegia CP [28] and another investigation included children with mixed type CP [23]. Regarding the level from the GMFCS, three studies included Levels I, II, and III [21,23,29,34], three others included Levels I and II [19,22,23,24,25,26,27,28,29,30,31,32,33,34,35]; three investigations included Levels III and IV [20,27,28]; one study included Levels I–V [17], one study Levels I–IV [22], another only Level III [30], and two investigations did not report the level from the GMFCS [26,31]. Finally, six investigations within their inclusion criteria considered the Manual Ability Classification System (MACS), including children with Levels I and II [29], Levels I, II, and III [18,21], Levels I–IV [22], and I–V [17]. One study did not describe the characteristics of the population included (Table 1).

### 3.4. Characteristics of the Intervention

The analysis of interventions recorded by the research studies included in this systematic review presented a high heterogeneity, where 11 interventions that used Nintendo Wii with a diversity of applications (Wii balance board, Wii sports resorts, Wii motion plus, etc.) stood out in the research [17,18,19,20,22,24,25,27,28,31,33], three with Kinect technology [21,29,35], one with Play Station controller immersion [20], and others with augmented reality activities [32,34,35], such as Biometrics Ltd. E-Link [26]. The average follow-up time of the groups was 2–12 weeks of intervention, with an average of 1–5 sessions per week according to the requirements of each research group. The sites for the applicability of the tests were spaces equipped for technology, such as hospitals, rehabilitation centers, and to a lesser extent, the individual’s home. The duration of the treatment ranged from 20 to 90 min on average.

**Figure 2:**
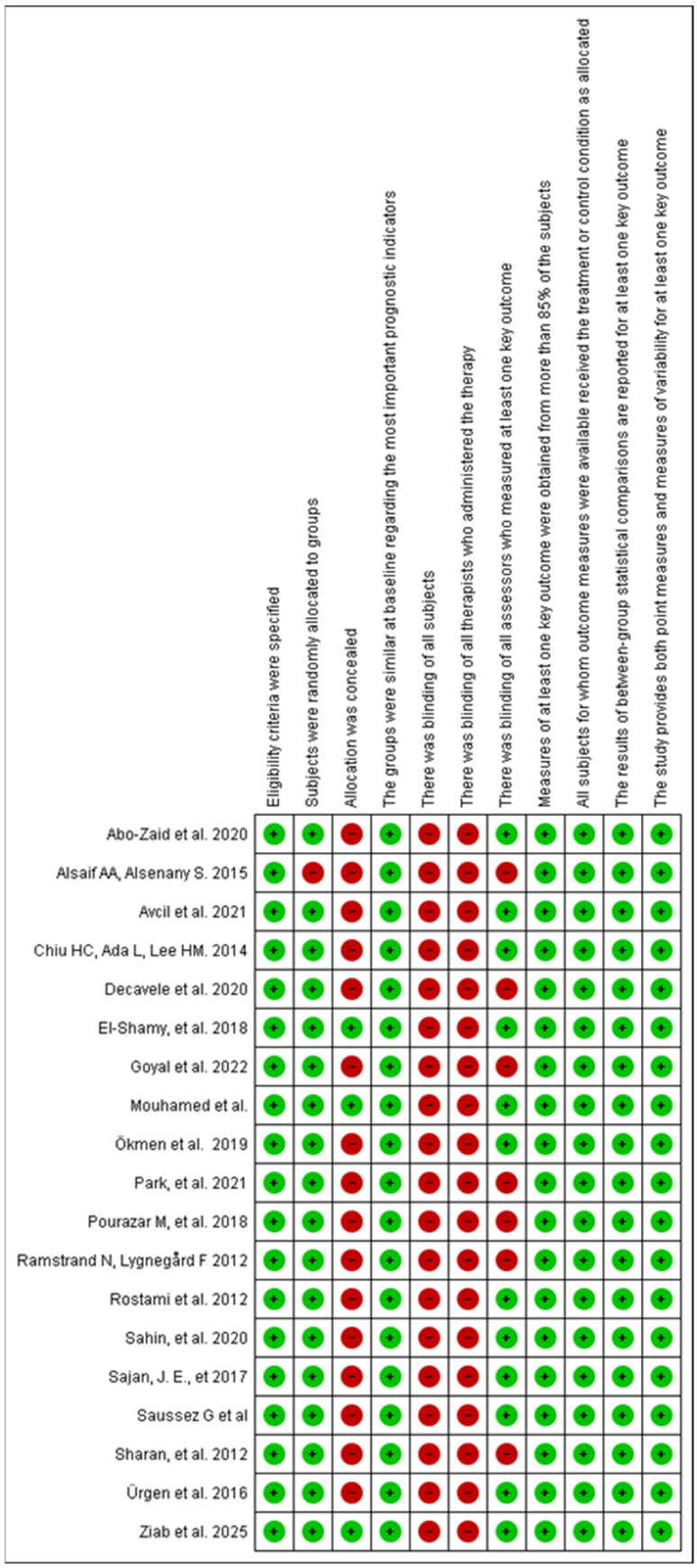
Summary of the risk of bias of the studies analyzed. Own source developed with RevMan 5.3. References: Abo-Zaid et al. (2021) [19], AlSaif AA, Alsenany S. (2015) [30], Avcil et al. (2021) [22], Chiu HC, Ada L, Lee HM. (2014) [17], Decavele et al. (2020) [20], El-Shamy SM et al. (2018) [18], Ökmen et al. (2019) [23], Park SH et al. (2021) [27], Pourazar et al. (2018) [29], Ramstrand N, Lygnegård F. (2012) [24], Rostami et al. (2012) [26], Şahin et al. (2020) [21], Sajan et al. (2017) [28], Sharan et al. (2012) [31], Ürgen et al. (2016) [25], Saussez G et al. (2023) [32], Mouhamed et al. (2024) [33], Goyal et al. (2022) [34], Ziab et al., 2025 [35].

The reliability of the results of the 19 controlled clinical trials was assessed to identify potential sources of bias in the trials (see Figure 2). Thus, 18 studies were found to perform an adequate randomization [17,18,19,20,21,22,23,24,25,26,27,28,29,31,32,33,34,35], 13 trials performed allocation concealment [16,17,18,19,20,21,22,23,24,25,26,27,28,29,31,33,35], 14 trials were classified as low risk for blinding of participants and therapists [17,18,19,20,21,22,23,24,25,26,27,28,30,31], nine studies provided complete information on blinding to outcome assessment [17,20,21,24,29,30,31,32,33,34,35], all the investigations described analyzed outcomes with complete information, and described the outcomes analyzed [17,18,19,20,21,22,23,24,25,26,27,28,29,30,31,32,33,34,35].

### 3.5. Quality of the Evidence

In order to describe the quality of the evidence of the studies, the GRADE approach was used, recognizing that the quality of the evidence can be categorized according to parameters given by the type of study design, the inconsistency of the results, and the way in which the direct or indirect evidence and imprecision was obtained. In this sense, an assessment was performed to determine how these factors affected the measures of results (see Table 2). Some outcomes were downgraded due to risk of bias when randomization or allocation concealment was not clearly described, or when incomplete outcome data suggested attrition bias (e.g., in studies assessing manual function with MMDT and JTTHF). Inconsistency was judged as serious when trials showed divergent results across studies, as occurred in some balance measures (PBS), where improvements varied in magnitude. Indirectness was considered in outcomes such as BOTMP, where the GMFCS was applied without clearly reporting how participants were distributed by severity levels. Imprecision was a frequent reason for downgrading, mainly due to small sample sizes and wide confidence intervals, as observed in outcomes like satisfaction and quality of life.

### 3.6. Effect of Interventions on Measures of Functionality

The following summarizes, by evaluation measure dimension, the scales used in the 19 studies included (Table 3).

#### 3.6.1. Motor Function

Motor function was assessed using the following scales: Gross Motor Function Measure (GMFM) [20,25,35], the Gross Motor Performance Measure (GMPM) [25], Pediatric Evaluation of Disability Inventory (PEDI) [25], Movement Assessment Battery for Children-2 (mABC-2) [30], Peabody Developmental Motor Scale (PMDS-2) [18], Bruininks–Oseretsky Test of Motor Proficiency (BOTMP) [26,30], and its short version (BOTMP-SF) [21], After applying VR interventions [18,22,23,27] found significant results for both groups [21,26], observed significantly greater improvements in the VR group [20,35], noted that the scores were significant in GMFM for the biped dimension, and [18] found no significant differences. Therefore, it appears that a positive effect is found on motor function following physical therapy intervention using VR in children with CP (Table 4).

#### 3.6.2. Balance

Regarding balance, this assessment measure was used with different tools, such as Pediatric Balance Scale (PBS) [20,25,28,31,33,35], Trunk Control Measurement Scale (TCMS) [27], Modified Functional Reach Test (mFRT) [27], Simple Reaction Time, Discriminant Reaction Time (DRT) [29], Static Posturography (PE), Timed up and go (TUG) [25], and Reactive Balance (BR) [24]. After applying VR there were significant differences in the balance of children with CP, with the following studies [20,25,27,29,31,35] versus two investigations where no significant differences were observed for any of the balance measures investigated [24,28,33]. According to the studies, it could be said that VR has a positive effect on the balance of children with CP (Table 4).

#### 3.6.3. Gait

Gait was assessed using 3D motion analysis (3DMA) [19], 1 min walk test (PC1 min) [30] 6 min walk [32], and Functional mobility scale (FMS) [19,23] reported statistically significant results in favor of the study group, especially for stride length, stride length, and cadence [19,30,33] also described changes after the treatment compared with pre-RV values (Table 4).

#### 3.6.4. Manual Function

Manual skills using Minnesota Manual Dexterity Test (MMDT) [22], Duruoz Hand Index (DEI) [22], Jebsen–Taylor Test of Hand Function (JTTHF) [17,32], MACS [33], Bimanual fine motor function (BFMF) [23] test, 9HPT [34] and the Quality of Upper Extremity Skills Test (QUEST) [28]. The results of the investigations suggest that there were significant positive results for manual skills in both groups [22,31,34]. The group in which VR was applied was statistically superior to the control group [22,23,28], whereas in the study by [17], there was no difference between the groups (Table 4).

#### 3.6.5. Quality of Life Measures

##### Activities and Participation

The level of participation and the execution of daily activities were evaluated in three investigations using the GMFCS [23], the WeeFunctional Independence Measure (WeeFIM) [21,34], and a survey prepared by the authors themselves to measure the level of participation [31]. The investigations concluded that there were statistically significant results in favor of the study group [21,23,31,34]. Hence, VR appears to exert a positive influence on the execution of activities and more effective participation for children with CP (Table 4).

##### Motivation and/or Satisfaction

Motivation and satisfaction after VR rehabilitation processes were analyzed in two investigations using the Mastery Motivation Questionnaire (DMQ) [20] and a motivation and satisfaction survey created by the authors [31]. According to [20], no significantly higher motivation was found after the application of VR, possibly because of the relatively simple design, the few games within the Openfeasyo Games platform, the lack of integration of special visual effects, and the simple graphic design. On the contrary, the level of motivation and satisfaction evaluated by [31] presented significantly higher results in the study group compared with the control group (Table 4).

##### Quality of Life

Quality of life was only evaluated in the research conducted by [22], describing significant changes in the Childhood Health Assessment Questionnaire sub scores for dressing, grasping, feeding, hygiene, reaching, and in the total score; however, there were no changes in the dimensions of lifting, gait, and activity. It should be noted that the objective of this research was to determine the effect of VR in the rehabilitation of the upper limbs of children with CP; therefore, the lower limbs were not worked out within the intervention protocols in both groups (Table 4).

Although the results regarding motor function, balance, gait, manual skills, activities, participation, motor learning, and quality of life generally suggest positive effects of VR interventions in children with CP, it is important to note that the certainty of the evidence was rated mostly as low or very low according to the GRADE assessment. This means that the improvements observed should be interpreted with caution, as they are not fully reliable and may change with stronger evidence. Moreover, while the GRADE approach was applied, a meta-analysis could not be performed due to the considerable heterogeneity across studies (differences in designs, interventions, durations, populations, and outcome measures). For this reason, causal claims in the conclusions have been tempered, emphasizing that the current findings should be considered promising but preliminary. This highlights the need for larger and better-designed randomized trials, with standardized assessment tools, to confirm these potential benefits.

## 4. Discussion

This systematic review synthesized the evidence on the use of virtual reality (VR) in children with cerebral palsy (CP) and its applications in health. Nineteen studies involving children with CP across various levels of the Gross Motor Function Classification System (GMFCS) were included in the analysis. All outcome measures showed positive changes following VR interventions. Among these, gross motor function and balance were the primary outcomes demonstrating the most significant improvements, with at least one of these two measures assessed in each study. These were followed by improvements in motivation and/or adherence. Positive changes were observed in two of the included studies [20,31]. Conversely, quality of life was addressed in studies [22,34], where VR interventions targeting upper extremity rehabilitation showed improvements in domains such as dressing, grip, feeding, hygiene, reaching, and overall functional scores.

From the point of view of implications for professional practice, it can be described that according to literature, the most widely used scale to evaluate the monitoring of gross motor function is the GMFM. However, of the total number of studies included in this review, only two investigations [20,25] used it as an evaluation measure, which still shows the great heterogeneity of the monitoring of goals for the functionality of children with CP by physical therapists. Regarding balance, the PBS was the most widely used measure by researchers to compare the VR interventions [20,25,28,31].

Likewise, an important limitation of the present review is the heterogeneity of the virtual reality interventions included, encompassing platforms such as Nintendo Wii, Xbox Kinect, PlayStation and other systems, without distinguishing between the different levels of interaction or immersion offered by each system. This lack of stratification limits the interpretation of the results, as the degree of immersion may influence both motor and cognitive engagement as well as therapeutic outcomes. It is recommended that future studies consider subgroup analyses based on the technological characteristics of the interventions, as this could clarify whether higher levels of immersion are associated with greater functional gains and improved adherence, thereby providing more precise recommendations for clinical practice.

On the other hand, regarding the VR application protocols, they were also diverse, with intervention durations ranging from 20 to 60 min, 3 to 6 times per week, over a period of 4 up to a maximum of 16 weeks.

### 4.1. Comparison with Other Reviews

This review provides an overview of the current context of virtual reality in cerebral palsy, considering the intervention without the addition of other interventions or combinations of therapies. The results obtained in this study indicate that there is an improvement at the clinical level in the main outcome measures. At the same time, variability was found in the instruments used in the evaluation processes, which prevents the establishment of final conclusions for inclusion in the neurorehabilitation processes in CP. These results are supported by Fandim et al. [5] and Ravi et al. [6], where evidence of low quality and moderate effect was found when adding VR to conventional rehabilitation. This was reflected in the improvement of the upper limb function, postural control, and balance and a small effect on the gait and lower limb strength measurements. Chen et al. [10] found a large effect size on arm function and postural control and a medium effect on ambulation, whereas Liu et al. [7] and Meneses et al. [36] found significant improvements in balance and gross motor function in children with CP.

Liu et al. (2022, 513 children) [7] and Komariah et al. (2024, ~900 participants) [37] reported significant benefits of virtual reality for balance and gross motor function, findings that were also observed in 10 and 6 studies of this synthesis, respectively. Improvements in daily functioning and independence [37], described as mixed by Liu et al. [7], were identified in 6 studies of the present review. Motivation and engagement, emphasized in the review by Luna-Oliva (2025, 616 children) [38], were also reported in 3 studies. Divergent findings emerged regarding upper limb outcomes: while da Silva (2021, 746 participants) [39] considered the evidence limited, 8 studies in this review demonstrated clear progress. Additionally, small but relevant reductions in spasticity (1–2 studies) were observed, an effect rarely addressed in previous analyses [39].

The review by Lopes et al. [3] observed that VR training, either alone or in combination with motor training, leads to improvements in sensorimotor functions and can be used as a supplement to other rehabilitation interventions, suggesting its use in future studies. A study by Warnier et al. [40] confirms this positive and promising effect on gait, which makes it a promising intervention for the rehabilitation of children with CP. However, these results should be interpreted with caution owing to the differences in the interventions used, the heterogeneity in the evaluations, and the relatively small size of the groups. In turn, Cano [41] found that VR has the potential to improve balance and gait, providing additional benefits when combined with conventional rehabilitation. Moreover, protocols should include principles of motor learning application to favor movement optimization processes. Amirthalinga et al. [42] and Alrashidi et al. [43] found that in the included studies on CP, there were no significant differences between the experimental and control groups.

In general, the adherence found in most of the studies and the motivational aspects obtained with this type of intervention should be highlighted. This level of adherence allows the inclusion of cognitive processes for the execution of motor tasks, which translate into the promising effects of the measures used. A review by Ul Ain et al. [44] concludes that VR work is effective and produces significant changes in the motor functions of patients with CP. Regarding executive functions, further research is needed to determine the impact of these games at the level of higher cognitive functions. A review conducted by Qien et al. [45] found that the use of VR could alleviate fatigue, tension, and depression and induce calmness and improve the quality of life in patients subjected to the intervention. This study, as well as the different reviews cited here, agrees on the need to include the use of validated assessment scales for motor function, gait, and balance.

The results of this study are aligned with those reported in studies using non immersive virtual reality, which also highlights meaningful changes in children’s social functioning. In those interventions, parents noted an increase in perceived social competence from 23.3 ± 9.6 to 30.0 ± 11.1 points, while children themselves reported gains from 31.3 ± 13.3 to 38.0 ± 12.4 points. Reductions in social anxiety, better global functioning, and more frequent initiation, response, and empathic behaviors were also described. Altogether, this body of evidence suggests that structured and repetitive digital practice can strengthen social interaction skills, complementing and supporting the progress—albeit more variable—observed in our participants [46].

### 4.2. Strengths and Weaknesses

Regarding the limitations of this review, the scarcity of clinical trials reduces the possibility of evaluating the available evidence on a large scale, although it is important to highlight that the total sample of all the studies included reached a representative sample of *n* = 704. Additionally, there is a high degree of heterogeneity regarding the population and intervention among the studies, meaning that the conclusions cannot be extrapolated to other contexts. The studies by Rostami et al. (2012) [26] and Sharan et al. (2012) [31] did not report the GMFCS levels of the participants included in their research. This lack of information may limit the interpretation of the present findings, as it hinders the ability to determine both the adequacy and the impact of the interventions on motor function levels when clear inclusion criteria are not provided. However, the methodological rigor and the evaluation of the evidence under the GRADE approach confers certainty upon the conclusions presented in this review.

Despite the above limitations, the importance of the use of VR in the therapeutic approach of children with CP is proven. Therefore, the importance of this type of intervention in neurorehabilitation protocols should be highlighted accordingly as well as the importance of having reliable and quantifiable tools to monitor the impact thereof in the functionality of the child.

### 4.3. Implications for Practice and Future Research

The results of this research demonstrate the importance of the use of VR within the physiotherapeutic alternatives in neurorehabilitation for children with CP. Therefore, it is considered relevant that these are included in the training curricula of physiotherapists as well as in the different fields of intervention within professional practice. Likewise, it is necessary that future research be oriented to identify the accessibility of the different VR equipment both from the telerehabilitation approach as well as from the face-to-face approach. It is also important to focus on the hospital, outpatient, and educational fields of action to determine the access opportunities for the general population.

Regarding the physiotherapeutic approach, it is necessary for the professionals to identify the level of functionality of children with CP and thus determine a therapeutic plan tailored to the needs of each child when applying VR to gain knowledge about their capabilities and strengths when selecting the equipment to be used. In this way, the levels of the GMFCS act as a guide for the approach of the intervention objectives and the VR therapeutic plan from physiotherapy.

In contrast with other earlier reviews that often-examined virtual reality in combination with other rehabilitation strategies or limited their focus to specific motor outcomes, our review looked at VR as a stand-alone intervention for children with cerebral palsy. This approach makes it possible to better understand its direct effects on gross motor function, balance, manual abilities, and motor learning.

The strength of this work lies in the broader time frame and larger evidence base considered: we included 19 randomized controlled trials published up to 2024, encompassing 704 participants, a substantially larger sample than those analyzed in reviews such as Fandim et al. [5] and Ravi et al. [6]. Importantly, we also addressed aspects of adherence and motivation, outcomes that have been less visible in prior syntheses but are highly relevant for clinical practice. By identifying where the evidence is strongest (gross motor function and balance) and where it remains more limited (manual skills and participation), this review provides a more nuanced perspective on the therapeutic role of VR and highlights directions for future research. Finally, future studies in the area should adhere to international methodological standards, such as CONSORT [47] and the checklist for TIDier interventions [48].

## 5. Conclusions

The findings of this study demonstrate clinically relevant improvements in key functional outcomes, including motor function and balance. While protocols incorporating the Nintendo Wii were most frequently employed, the heterogeneity of assessment instruments across studies limits the ability to draw definitive conclusions. Furthermore, the predominance of low to very low certainty evidence underscores the need for caution in interpretation and restricts the strength of generalizations. Nevertheless, the current evidence suggests that such tools hold promise for integration into neurorehabilitation programs for individuals with cerebral palsy. Future studies should prioritize the standardization of outcome measures and the implementation of larger, high-quality randomized controlled trials. Importantly, the high levels of adherence associated with these interventions represent a valuable asset from a clinical perspective.

## Figures and Tables

**Table 1:** Consolidated articles included in the systematic review.

Study	Population	Age (Years)	GMFCS	RV Intervention	Group Control	Duration	Place
Abo-Zaid et al. (2021) [19]	60 children with hemiplegic CP	8–12	I–II	Nintendo Wii—Nintendo Co., Ltd., Kioto (Kyoto), Japan	NDT	60 min/3 days/16 weeks	Clinical setting
AlSaif AA, Alsenany S. (2015) [30]	40 children with spastic diplegia CP	6–10	III	Nintendo Wii—Nintendo Co., Ltd., Kioto (Kyoto), Japan	Not reported	20 min/1 day/12 weeks	At home
Avcil et al. (2021) [22]	30 children with CP hemiplegia, diplegia, and dyskinetic type	8–12	Range I–IV	Nintendo Wii—Nintendo Co., Ltd., Kioto (Kyoto), Japan	NDT-based upper extremity rehabilitation	20 min/3 days/8 weeks	Clinical setting
Chiu HC, Ada L, Lee HM. (2014) [17]	62 children with hemiparesis CP	5–13	Range I–V	Nintendo Wii—Nintendo Co., Ltd., Kioto (Kyoto), Japan	Conventional physiotherapy	3 days/6 weeks	At home
Decavele et al. (2020) [20]	32 children with hemiparesis CP	6–15	III–IV	Nintendo Wii and Xbox Kinect—Nintendo Co., Ltd., Kioto (Kyoto), Japan/Microsoft Corporation, Redmond, WA, USA	Conventional physiotherapy	45 min/2 days/12 weeks	Rehabilitation center
El-Shamy SM et al. (2018) [18]	40 children with hemiparesis CP	8–12	Range I–III	Nintendo Wii—Nintendo Co., Ltd., Kioto (Kyoto), Japan	Conventional physiotherapy	60 min/3 days/12 weeks	Clinical setting
Ökmen et al. (2019) [23]	41 children with hemiplegia, diplegia, quadriplegia, dyskinetic and mixed CP	5–15	Range I–III	PlayStation—Sony Interactive Entertainment Inc., San Mateo, CA, USA	NDT and conventional physiotherapy	60 min/3 days/4 weeks	Clinical setting
Park SH et al. (2021) [27]	20 children with diplegia and spastic quadriplegia CP	6–18	III–IV	Nintendo Wii—Nintendo Co., Ltd., Kioto (Kyoto), Japan	Arm reach training in the sitting position	40 min/2 days/4 weeks	Clinical setting
Pourazar et al. (2018) [29]	30 children with CP.	7–12	Range I–III	Xbox Kinect—Microsoft Corporation, Redmond, WA, USA	Conventional physiotherapy	20 sessions/6 weeks	Rehabilitation center
Ramstrand N, Lygnegård F. (2012) [24]	18 children with hemiplegic or diplegic CP	8–17	I–II	Nintendo Wii—Nintendo Co., Ltd., Kioto (Kyoto), Japan	Without intervention	30 min/5 days/5 weeks	At home
Rostami et al. (2012) [26]	32 children with spastic hemiparesis CP	6–11	Does not report.	Biometrics Ltd. E-Link—Biometrics Ltd., Gwent, UK	Group CIMT, Group combined VR and modified CIMT, Group control (conventional therapy)	90 min/3 days/4 weeks	Rehabilitation center
Şahin et al. (2020) [21]	60 children with spastic hemiparesis CP	7–16	I–II	Xbox Kinect—Microsoft Corporation, Redmond, WA, USA	NDT	45 min/2 days/8 weeks	Clinical setting
Sajan et al. (2017) [28]	20 children with CP	5–20	I–IV	Nintendo Wii—Nintendo Co., Ltd., Kioto (Kyoto), Japan	Conventional therapy	45 min/6 days/3 weeks	Clinical setting
Sharan et al. (2012) [31]	29 children with CP	8–15	Does not report.	Nintendo Wii—Nintendo Co., Ltd., Kioto (Kyoto), Japan	Not reported	3 days/3 weeks	At home
Ürgen et al. (2016) [25]	30 children with spastic hemiplegic CP	7–14	I–II	Nintendo Wii—Nintendo Co., Ltd., Kioto (Kyoto), Japan	Not reported	45 min/2 days/9 weeks	Rehabilitation center
Saussez G et al. (2023) [32]	40 children with CP	5–18	I–II	REAtouch®—Mobility Research Inc. (LiteGait), Tempe, AZ, USA	HABIT-ILE	90 h spread over 10–12 consecutive days.	HABIT-ILE camps
Mouhamed et al. (2024) [33]	64 children with ataxic cerebral palsy	9–14	I–II	Nintendo Wii (balance board)—Nintendo Co., Ltd., Kioto (Kyoto), Japan	Conventional physiotherapy	3 sessions/week for 3 months (60 min/session)	Clinical setting
Goyal et al. (2022) [34]	10 unilateral CP	6–10	Range I–III	NIVR—Non-Immersive Virtual Reality (generic term)	Occupational therapy	5 sessions per week for 4 weeks (20 sessions)	Clinical setting
Ziab et al., 2025 [35]	46 children with CP	4–12	I–II	Xbox Kinect—Microsoft Corporation, Redmond, WA, USA	Conventional physiotherapy	18 sessions (3/week, 6 weeks)	Rehabilitation Centers

NDT, Neurodevelopment Therapy; CIMT, Constraint-Induced Movement Therapy; NIVR, Non-immersive virtual reality, REAtouch^®^, virtual device; HABIT-ILE, Hand-Arm Bimanual Intensive Therapy Including Lower Extremities.

**Table 2:** Summary evaluation of the quality of evidence of the studies.

Certainty Evaluation						
№ de Studies	Study Design	Risk of Bias	Inconsistency	Indirect Evidence	Inaccuracy	Quality of Evidence
Gross Motor Function measured with GMFM						
3	randomized trials	not serious	not serious	not serious	serious ^a^	Low
Gross Motor Function measured with BOTMP						
2	randomized trials	serious ^b^	not serious	serious ^c^	serious ^a^	Very Low
Gross Motor Function measured with GMPM, PEDI, mABC-2, BOTMP-SF and PMDS-2						
4	randomized trials	serious ^b^	not serious	not serious	serious ^a^	Low
Balance measured with PBS						
4	randomized trials	not serious	serious ^d^	not serious ^a^	serious ^a^	Low
Balance measured with TCMS, mFRT, SRT, DRT, BR, PE and TUG						
6	randomized trials	not serious	not serious	not serious	serious ^a^	Low
March measured with 3DMA, PC1 min, FMS						
3	randomized trials	serious ^b^	not serious	not serious	serious ^a^	Low
Manual Function measured with MMDT, DEI, JTTHF, MACS, BFMF, QUEST						
5	randomized trials	serious ^e^	serious ^d^	not serious	serious ^a^	Very Low
Motivation and/or satisfaction measured with DMQ and level of motivation-satisfaction						
2	randomized trials	serious ^a^	serious ^d^	not serious	serious ^a^	Very Low
Activities and participation measured with GMFCS, WeeFim and level of participation						
3	randomized trials	serious ^a^	serious ^d^	not serious	serious ^a^	Very Low
Quality of life measured with CHAQ						
1	randomized trials	not serious	not serious	not serious	serious ^a^	Low

Explanations: a. Small samples with an ample CI (confidence interval). b. Risk of selection bias related to allocation and concealment. c. GMFCS was applied without identifying how the groups were distributed in one of the studies. d. Small sample size with a substantial loss of participants. e. There is a risk of attrition related to the presentation of incomplete results ≍ ⨁.

**Table 3:** Evaluation measures by dimensions of the included studies.

Dimension/Domain	Instruments/Measures Used
Motor function	Gross Motor Function Measure (GMFM) [20,25,35] Gross Motor Performance Measure (GMPM) [25] Pediatric Evaluation of Disability Inventory (PEDI) [25] Movement Assessment Battery for Children-2 (mABC-2) [30] Peabody Developmental Motor Scale (PDMS-2) [18] Bruininks–Oseretsky Test of Motor Proficiency (BOTMP) [26,30] BOTMP Short Form (BOTMP-SF) [21]
Balance	Pediatric Balance Scale (PBS) [20,25,28,31,33,35] Trunk Control Measurement Scale (TCMS) [27] Modified Functional Reach Test (mFRT) [27] Simple Reaction Time, Discriminant Reaction Time (DRT) [28] Static Posturography (PE) [26] Timed Up and Go (TUG) [25] Reactive Balance (BR) [24]
Gait/Ambulation	3D Motion Analysis (3DMA) [19] 1 min Walk Test [30] 6 min Walk Test [32] Functional Mobility Scale (FMS) [19,23]
Manual skills/Manual Function	Minnesota Manual Dexterity Test (MMDT) [22] Duruöz Hand Index (DHI) [22] Jebsen–Taylor Test of Hand Function (JTTHF) [17,32] MACS (Manual Ability Classification System) [33] Bimanual Fine Motor Function (BFMF) [23] 9-Hole Peg Test (9HPT) [34] Quality of Upper Extremity Skills Test (QUEST) [28]
Activities/Participation/Quality of Life	GMFCS (Gross Motor Function Classification System) [23] WeeFIM (Wee Functional Independence Measure) [21,34] Childhood Health Assessment Questionnaire [22]
Motivation/Satisfaction	Mastery Motivation Questionnaire (DMQ) [20] Author-designed motivation/satisfaction survey [31].

**Table 4:** Consolidated results and interventions conducted in the studies included in the systematic review.

Study	Outcome Measures	Main Results
Abo-Zaid et al. (2021) [19]	3DMA	Swing Phase (%) Pre G1: 47.08 ± 1.99 Post: G1: 44.92 ± 1. Pre: G2: 47.38 ± 2.20 Post: G2: 45.87 ± 1.50 Cadence (steps/min) Pre G1: 83.91 ± 3.621 Post G1: 87.13 ± 3.025 Pre G2: 82.72 ± 4.874 Post G2: 84.40 ± 3.965
AlSaif AA, Alsenany S. (2015) [30]	mABC-2, BOTMP, PC1 min Walk test	mABC-2 Pre G1: 38.3 ± 5.42 Post G1: 44.1 ± 5.21 Pre G2: 38.9 ± 5.27 Post G2: 39.1 ± 5.16 BOTMP Pre G1: 2.23 ± 0.47 Post G1: 3.78 ± 0.39 Pre G2: 2.82 ± 0.51 Post G2: 3.12 ± 0.66 1 min WT Pre G1: 90.1 ± 7.21 Post G1: 98.8 ± 6.75 Pre G2: 91.1 ± 6.93 Post G2: 91.8 ± 6.82
Avcil et al. (2021) [22]	MMDT, CHAQ, DEI, DM	MMDT Pre G1: 508.82 ± 303.34 Post G1: 346.53 ± 188.19 Pre G2: 450.93 ± 447.51 Post G2: 385.73 ± 412.27 CHAQ Pre G1: 1.70 ± 0.57 Post G1: 1.37 ± 0.54 Pre G2: 2.31 ± 0.67 Post G2: 1.87 ± 0.68
Chiu HC, Ada L, Lee HM. (2014) [17]	Coordination, 9HPT, JTTHF	Coordination Pre G1: 0.26 ± 0.18 Post G1: 0.28 ± 0.20 Pre G2: 0.30 ± 0.22 Post G2: 0.28 ± 0.21 9HPT Pre G1: 0.10 ± 0.09 Post G1: 0.12 ± 0.10 Pre G2: 0.12 ± 0.10 Post G2: 0.13 ± 0.11 JTTHF Pre G1: 0.21 ± 0.14 Post G1: 0.26 ± 0.18 Pre G2: 0.22 ± 0.15 Post G2: 0.27 ± 0.20
Decavele et al. (2020) [20]	GAS, TCMS, PBS, GMFM-88, DMQ	GAS Pre G1: 29.9 Post G1: 38.4 *p* =< 0.001 Pre G1: 27.9 Post G1: 30.0 *p* = 0.007 TCMS Pre G1: 37.8 Post G1: 42.3 *p* =< 0.001 Pre G2: 35.1 Post G2: 33.1 *p* = 0.124 GMFM Pre G1: 52.9 Post G1: 54.4 *p* =< 0.001 Pre G2: 45.0 Post G2: 44.1 *p* = 0.317 PBS Pre G1: 22.8 Post G1: 24.1 *p* = 0.01 Pre G2: 18.9 Post G2: 18.5 *p* = 0.264 DMQ G1: 76.8 G2: 79.8
El-Shamy SM et al. (2018) [18]	MAS, DM, PMDS-2	MAS Pre G1: 2.5 ± 0.6 Post G1: 1.6 ± 0.3 DM Pre G1: 8.7 ± 1.9 Post G1: 11 ± 1.5 Pre G2: 8.5 ± 1.6 Post G2: 9.2 ± 1.2 PMDS-2 Pre G1: 3 Post G1: 3 Pre G2: 2 Post G2: 2
Ökmen et al. (2019) [23]	BFMF, GMFCS, FMS	BFMF Pre G1: 3 Post G1: 2 *p* = 0.001 * Pre G2: 2.5 Post G2: 2.5 *p* = 0.317 GMFCS Pre G1: 3 Post G1: 3 *p* = 0.005 * Pre G2: 3 Post G2: 3 FMS Pre G1: 2 Post G1: 2.5 *p* = 0.003 * Pre G2: 3 Post G2: 3 *p* = 0.17
Park SH et al. (2021) [27]	Software Wii Balance Board, mFRT, TCMS	Velocity Pre G1: 7.25 ± 4.99 (cm/s) Post G1: 5.68 ± 4.51 *p* = 0.005 Pre G2: 6.78 ± 5.19 Post G2: 6.41 ± 5.21 *p* = 0.169 mFRT: Pre G1: 16.02 ± 4.52 Post G1: 21.05 ± 6.34 *p* = 0.005 TCMS Pre G1: Pre 19.5 ± 9.67 Post G1: 27.4 ± 10.86 *p* = 0.005 Pre G2: 17.3 ± 7.7 Post G2: 22.3 ± 9.17 *p* = 0.005
Pourazar et al. (2018) [29]	SRT y DRT	SRT Pre G1: 0.605 ± 0.133 Post G1: 0.343 ± 0.156 Pre G2: 0.610 ± 0.265 Post G2: 0.576 ± 0.236 DRT Pre G1: 0.936 ± 0.292 Post G1: 0.568 ± 0.177 Pre G2: 0.952 ± 0.289 Post G2: 0.884 ± 0.343
Ramstrand N, Lygnegård F. (2012) [24]	mSOt y BR	Stable support (eyes open) Pre G1: 2.34 Post G1: 2.21 Pre G2: 2.34 Post G2: 2.17 Stable support (eyes closed) Pre G1: 2.70 Post G1: 2.59 Pre G2: 2.70 Post G2: 2.59
Rostami et al. (2012) [26]	BOTMP	Amount of use Pre G1: 0.66 ± 0.37 Post G1: 2.37 ± 0.45 Pre G2: 0.74 ± 0.24 Post G2: 2.54 ± 0.51 Quality of movement Pre G1: 0.53 ± 0.31 Post G1: 2.26 ± 0.24 Pre G2: 0.59 ± 0.28 Post G2: 2.21 ± 0.19
Şahin et al. (2020) [21]	BOTMP-SF y WeeFIM	BOTMP-SF Pre G1: 35.46 ± 20.29 Post G1: 69.96 ± 34.42 0.0001 * Pre G2: 36.36 ± 19.83 45.10 ± 18.14 0.028 * WeeFIM Pre G1: 103.06 ± 15.04 Post G1: 112.96 ± 10.20 0.0001 * Pre G2: 102.56 ± 14.88 Post G2: 104.70 ± 13.67 0.012 *
Sajan et al. (2017) [28]	PE, PBS, QUEST, BBT, TVPS-3.	Sway velocity eyes open (mm/s) Pre G1: 137.67 ± 179.52 Post G1: 83.06 ± 67.73 Pre G2: 137.66 ± 96.06 Post G2: 127.93 ± 86.33 PBS Pre G1: 15.70 ± 14.77 Post G1: 18.7 ± 16.33 Pre G2: 20.44 ± 19.1 Post G2: 25.00 ± 17.71 BBT Pre G1: 46.9 ± 16.39 Post G1: 55.2 ± 19.70 Pre G2: 59.33 ± 27.77 Post G2: 68.00 ± 27.09 QUEST Pre G1: 72.86 ± 22.57 Post G1: 76.38 ± 19.72 Pre G2: 86.83 ± 9.78 Post G2: 88.45 ± 9.30 TVPS: Pre G1: 32.10 ± 9.60 Post G1: 36.50 ± 9.63 Pre G2: 37.30 ± 13.20 Post G2: 43.44 ± 14.93 Walk Pre G1: 12.61 ± 19.73 Post G1: 20.61 ± 28.42 Pre G2: 23.89 ± 33.23 Post G2: 34.31 ± 39.98
Sharan et al. (2012) [31]	MACS, PBS, level of participation, motivation, and child satisfaction.	MACS: Pre G1: 1.71 ± 0.99 Post G1: 1.43 ± 0.65 *p* < 0.05 * Pre G2: 2.20 ± 1.21 Post G2: 1.73 ± 0.80 *p* < 0.01 * PBS: Pre G1: 35.57 ± 12.67 Post G1: 45.00 ± 8.73 *p* < 0.001 * Pre G2: 26.40 ± 14.63 Post G2: 36.07 ± 14.38 *p* < 0.001 *
Ürgen et al. (2016) [25]	GMFM, GMPM, TUG, PBS, PEDI	GMFM: Pre G1: 91.89 ± 3.80 Post G1: 96.05 ± 2.43 Pre G2: 87.95 ± 6.04 Post G2: 90.92 ± 5.04 GMPM Pre G1: 197.20 ± 15.42 Post G1: 206.00 ± 15.51 Pre G2: 185.13 ± 24.89 Post G2: 190.40 ± 22.65 TUG Pre G1: 6.48 ± 0.85 Post G1: 6.26 ± 0.67 Pre G1: 6.60 ± 0.82 Post G1: 6.55 ± 0.71 PBS Pre G1: 50.07 ± 2.86 Post G1: 53.80 ± 1.61 Pre G2: 48.47 ± 3.50 Post G2: 49.27 ± 3.12 PEDI G1: 180.07 ± 12.36 G2: 181.73 ± 8.18
Saussez G et al. (2023) [32]	AHA, JTTHF, BBT, MFPT, 6MW, ABILHAND-Kids, ACTIVLIM-CP, PEDI, ABILOCO-Kids, COPM.	AHA Pre G1: 54.9 ± 18 Post G1: 58.4 ± 29 *p* = 0.002 Pre G2: 58.3 ± 16 Post G2: 60.3 ± 16 *p* = 0.039 BBT Pre G1: 20.8 ± 13 Post G1: 21.7 ± 13 *p* = 0.092 Pre G2: 22 ± 11 Post G2: 23.1 ± 11 *p* = 0.029 JTTHF Pre G1: 419 ± 358 Post G1: 364 ± 333 Pre G2: 412 ± 344 Post G2: 367 ± 344 6MWT Pre G1: 467 ± 90 469 ± 101 Post G1: 469 ± 101 Pre G2: 478 ± 106 Post G2: 479 ± 117
Mouhamed et al. (2024) [33]	PBS, OASI, APSI, MLSI	PBS: Pre G1: 22.34 ± 3.21 Post G1: 41.38 ± 2.72 Pre G2: 23.78 ± 3.89 Post G2: 36.03 ± 2.85 OASI Pre G1: 5.40 ± 0.56 Post G1: 3.32 ± 0.77 Pre G2: 5.55 ± 0.47 Post G2: 4.36 ± 0.64 APSI Pre G1: 5.21 ± 0.55 Post G1: 3.47 ± 0.36 Pre G2: 5.13 ± 0.58 Post G2: 3.76 ± 0.38 MLSI Pre G1: 4.60 ± 0.35 Post G1: 3.04 ± 0.42
Goyal et al. (2022) [34]	9HPT, BBT, ABILHAND-Kids, WeeFIM	9HPT Pre G1: 55.80 ± 6.01 Post G1: 39.80 ± 4.43 Pre G2: 56.80 ± 7.19 Post G2: 51.40 ± 6.58 BBT Pre G1: 15.60 ± 3.50 Post G1: 26.60 ± 2.30 Pre G2: 14.00 ± 3.53 Post G2: 17.80 ± 5.01 ABILHAND Kids Pre G1: 50.40 ± 6.54 Post G1: 64.00 ± 3.00 Pre G2: 44.40 ± 7.36 Post G2: 47.80 ± 5.93 WeeFIM Pre G1: 28.60 ± 7.36 Post G1: 35.40 ± 7.23 Pre G2: 25.80 ± 5.80 Post G2: 27.20 ± 5.16
Ziab et al., 2025 [35]	GMFM (D y E), PBS, FTSTST, COM (UCOM y LCOM)	GMFM (D) G1: 6.50 *p* = 0.000 G2: −1.40 *p* = 0.01 GMFM (E G1: −7.07P= 0.000 G2: −2.40 *p* = 0.000 PBBS: G1: −9.14 *p* = 0.000 G2: −0.87P= 0.000 FTSTST G1: 9.21 *p* = 0.000 G2: 1.40 *p* = 0.000 LUCOM G1: 6.68 *p* = 0.000 G2: 0.26 *p* = 0.25

3DMA, 3D Motion Analysis; mABC-2, movement Assessment Battery for Children-2; BOTMP, Bruininks–Oseretsky Test of Motor Proficiency; PC1 min, 1 min walk test; MMDT, Minnesota Manual Dexterity Test; CHAQ, Childhood Health Assessment Questionnaire; DEI, Duruoz Hand Index; DM, Dynamometer; 9HPT, Nine Hole Peg Test, JTTHF, Jebsen–Taylor Test of Hand Function; GAS, Goal Attainment Scale; TCMS, Trunk Control Measurement Scale; PBS, Pediatric Balance Scale; GMFM-88, Gross Motor Function Measure-88; DMQ, Mastery Motivation Questionnaire; MAS, Modified Ashworth Scale; PMDS-2, Peabody Developmental Motor Scale; BFMF, Bimanual Fine Motor Function; GMFCS, Gross Motor Function Classification System levels; FMS, Functional Mobility Scale; mFRT, modified Functional Reach Test; SRT, Simple Reaction Time; DRT, Discriminatory Reaction Time; mSOT, modified Sensory Organization Test; BR, balance reactive; BOTMP-SF, Bruininks–Oseretsky Test of Motor Proficiency-Short Form; WeeFIM, WeeFunctional Independence Measure; PE, Posturografía estática; QUEST, Quality of Upper Extremity Skills Test; BBT, Box and Block Test; TVPS-3, Test for Visual-Perceptual Skills, third edition; MACS, Manual Ability Classification System; TUG, Timed up and go; GMPM, the Gross Motor Performance Measure; PEDI, Pediatric Evaluation of Disability Inventory, AHA, Assisting Hand Assessment; BBT, Box and Blocks Test; MFPT, Manual Form Perception Test; 6MW, 6 Minute Walk Test; COPM, Canadian Occupational Performance Measure; OASI, Overall Stability Index; APSI, Anteroposterior Stability Index; FTSTST, Five Times Sit-To-Stand Test, COM, center of mass, UCOM, Upper center of mass, LCOM, Lower center of mass, * statistical significance.

## Data Availability

No new data were created or analyzed in this study. Data sharing is not applicable to this article.

## Appendix A. Search Strategies

MEDLINE (via PubMed): (“cerebral palsy” [MeSH Terms] OR “cerebral palsy” [Title/Abstract]) AND (“physical therapy modalities” [MeSH Terms] OR “physical therapy” [Title/Abstract] OR “physiotherapy” [Title/Abstract]) AND (“virtual reality” [MeSH Terms] OR “virtual reality” [Title/Abstract])

LILACS: “cerebral palsy” AND (“physical therapy” OR “physiotherapy”) AND “virtual reality”

ScienceDirect: TITLE-ABSTR-KEY (“cerebral palsy” AND (“physical therapy” OR “physiotherapy”) AND “virtual reality”)

PEDro: Abstract & Title: cerebral palsy AND physical therapy OR physiotherapy AND virtual reality

SpringerLink: “cerebral palsy” AND (“physical therapy” OR “physiotherapy”) AND “virtual reality”

Cochrane Library: (“cerebral palsy” in Title Abstract Keyword) AND (“physical therapy” OR “physiotherapy” in Title Abstract Keyword) AND (“virtual reality” in Title Abstract Keyword)

EBSCOhost (Academic Search Complete/CINAHL): (cerebral palsy) AND (physical therapy OR physiotherapy) AND (virtual reality) Search fields: Title, Abstract, Subject Terms

Google Scholar: “cerebral palsy” “physical therapy” OR “physiotherapy” “virtual reality”

## References

[1] Albesa S.A., Nova Díaz D.M., Aznal Sáinz E. (2023). Cerebral Palsy: New Challenges in the Era of Rare Diseases. An. Sist. Sanit. Navar..

[2] Park M.S., Kim S.J., Chung C.Y., Kwon D.G., Choi I.H., Lee K.M. (2011). Prevalence and Lifetime Healthcare Cost of Cerebral Palsy in South Korea. Health Policy.

[3] Lopes J.B.P., Duarte N.A.C., Lazzari R.D., Oliveira C.S. (2020). Virtual Reality in the Rehabilitation Process for Individuals with Cerebral Palsy and Down Syndrome: A Systematic Review. J. Bodyw. Mov. Ther..

[4] Perez-Marcos D. (2018). Virtual Reality Experiences, Embodiment, Videogames and Their Dimensions in Neurorehabilitation. J. Neuroeng. Rehabil..

[5] Fandim J.V., Saragiotto B.T., Porfírio G.J.M., Santana R.F. (2021). Effectiveness of Virtual Reality in Children and Young Adults with Cerebral Palsy: A Systematic Review of Randomized Controlled Trial. Braz. J. Phys. Ther..

[6] Ravi D.K., Kumar N., Singhi P. (2017). Effectiveness of Virtual Reality Rehabilitation for Children and Adolescents with Cerebral Palsy: An Updated Evidence-Based Systematic Review. Physiotherapy.

[7] Liu C., Wang X., Chen R., Zhang J. (2022). The Effects of Virtual Reality Training on Balance, Gross Motor Function, and Daily Living Ability in Children with Cerebral Palsy: Systematic Review and Meta-Analysis. JMIR Serious Games.

[8] World Health Organization (WHO) (2001). International Classification of Functioning, Disability and Health: ICF: Short Version. https://apps.who.int/iris/handle/10665/43360.

[9] Ministerio de Salud (MinSalud) (2019). Modelo de Acción Integral Territorial (MAITE). https://minsalud.gov.co/sites/rid/Lists/BibliotecaDigital/RIDE/DE/DIJ/resolucion-2626-de-2019.pdf?ID=20138.

[10] Chen Y., Fanchiang H.C.D., Howard A. (2018). Effectiveness of Virtual Reality in Children with Cerebral Palsy: A Systematic Review and Meta-Analysis of Randomized Controlled Trials. Phys. Ther..

[11] Levac D., Huber M.E., Sternad D. (2021). Learning and Transfer of Complex Motor Skills in Virtual Reality: Implications for Pediatric Rehabilitation. Phys. Ther..

[12] Fang E., Guan H., Du B., Ma X., Ma L. (2025). Effectiveness of virtual reality for functional disorders in cerebral palsy: An overview of systematic reviews and meta-analyses. Front. Neurol..

[13] Hao J., Huang B., Remis A., He Z. (2023). The Application of Virtual Reality to Home-Based Rehabilitation for Children and Adolescents with Cerebral Palsy: A Systematic Review and Meta-Analysis. Physiother. Theory Pract..

[14] Page M.J., McKenzie J.E., Bossuyt P.M., Boutron I., Hoffmann T.C., Mulrow C.D., Shamseer L., Tetzlaff J.M., Akl E.A., Brennan S.E. (2021). The PRISMA 2020 Statement: An Updated Guideline for Reporting Systematic Reviews. BMJ.

[15] Maher C.G., Sherrington C., Herbert R.D., Moseley A.M., Elkins M. (2003). Reliability of the PEDro Scale for Rating Quality of Randomized Controlled Trials. Phys. Ther..

[16] Schünemann H., Brożek J., Guyatt G., Oxman A. (2013). GRADE Handbook for Grading Quality of Evidence and Strength of Recommendations.

[17] Chiu H.C., Ada L., Lee H.M. (2014). Upper Limb Training Using Wii Sports Resort for Children with Hemiplegic Cerebral Palsy: A Randomized, Single-Blind Trial. Clin. Rehabil..

[18] El-Shamy S.M., El-Banna M.F. (2020). Effect of Wii Training on Hand Function in Children with Hemiplegic Cerebral Palsy. Physiother. Theory Pract..

[19] Abo-Zaid N.A., Helmy N.A., Elsayed N.I., Mohammed A.H. (2021). Wii Sport versus Task-Oriented Training on Gait in Unilateral Cerebral Palsy: A Randomized Controlled Trial. J. Hum. Sport Exerc..

[20] Decavele S., Ortibus E., Van Campenhout A., Molenaers G., Jansen B., Omelina L., Franki I. (2020). The Effect of a Rehabilitation Specific Gaming Software Platform to Achieve Individual Physiotherapy Goals in Children with Severe Spastic Cerebral Palsy: A Randomized Crossover Trial. Games Health J..

[21] Şahin S., Köse B., Aran O.T., Bahadlr Aǧce Z., Kaylhan H. (2020). The Effects of Virtual Reality on Motor Functions and Daily Life Activities in Unilateral Spastic Cerebral Palsy: A Single-Blind Randomized Controlled Trial. Games Health J..

[22] Avcil E., Tarakci D., Arman N., Tarakci E. (2021). Upper Extremity Rehabilitation Using Video Games in Cerebral Palsy: A Randomized Clinical Trial. Acta Neurol. Belg..

[23] Ökmen B.M., Aslan M.D., Nakipoğlu Yüzer G.F., Özgirgin N. (2019). Effect of Virtual Reality Therapy on Functional Development in Children with Cerebral Palsy: A Single-Blind, Prospective, Randomized-Controlled Study. Turk. J. Phys. Med. Rehabil..

[24] Ramstrand N., Lygnegård F. (2012). Can Balance in Children with Cerebral Palsy Improve Through Use of an Activity Promoting Computer Game?. Technol. Health Care.

[25] Urgen M., Akbayrak T., Gunel M., Cankaya O., Guchan Z., Turkyilmaz E. (2016). Investigation of the Effects of the Nintendo Wii-Fit Training on Balance and Advanced Motor Performance in Children with Spastic Hemiplegic Cerebral Palsy: A Randomized Controlled Trial. Int. J. Ther. Rehabil. Res..

[26] Rostami H.R., Arastoo A.A., Nejad S.J., Mahany M.K., Malamiri R.A., Goharpey S. (2012). Effects of Modified Constraint-Induced Movement Therapy in Virtual Environment on Upper-Limb Function in Children with Spastic Hemiparetic Cerebral Palsy: A Randomised Controlled Trial. NeuroRehabilitation.

[27] Park S.H., Son S.M., Choi J.Y. (2021). Effect of Posture Control Training Using Virtual Reality Program on Sitting Balance and Trunk Stability in Children with Cerebral Palsy. NeuroRehabilitation.

[28] Sajan J.E., John J.A., Grace P., Sabu S.S., Tharion G. (2017). Wii-Based Interactive Video Games as a Supplement to Conventional Therapy for Rehabilitation of Children with Cerebral Palsy: A Pilot, Randomized Controlled Trial. Dev. Neurorehabilit..

[29] Pourazar M., Mirakhori F., Hemayattalab R., Bagherzadeh F. (2018). Use of Virtual Reality Intervention to Improve Reaction Time in Children with Cerebral Palsy: A Randomized Controlled Trial. Dev. Neurorehabilit..

[30] Alsaif A.A., Alsenany S. (2015). Effects of Interactive Games on Motor Performance in Children with Spastic Cerebral Palsy. J. Phys. Ther. Sci..

[31] Sharan D., Ajeesh P.S., Rameshkumar R., Mathankumar M., Paulina R.J., Manjula M. (2012). Virtual Reality-Based Therapy for Postoperative Rehabilitation of Children with Cerebral Palsy. Work.

[32] Saussez G., Bailly R., Araneda R., Paradis J., Ebner-Karestinos D., Klöcker A., Sogbossi E.S., Riquelme I., Brochard S., Bleyenheuft Y. (2023). Efficacy of Integrating a Semi-Immersive Virtual Device in the HABIT-ILE Intervention for Children with Unilateral Cerebral Palsy: A Non-Inferiority Randomized Controlled Trial. J. Neuroeng. Rehabil..

[33] Mouhamed H.A., Abo-Zaid N.A., Khalifa H.A., Ali M.E., Elserty N.S., Behiry M.A., Heneidy W.E. (2024). Efficacy of Virtual Reality on Balance Impairment in Ataxic Cerebral Palsy Children: Randomized Controlled Trial. Eur. J. Phys. Rehabil. Med..

[34] Goyal C., Vardhan V., Naqvi W. (2022). Non-Immersive Virtual Reality as an Intervention for Improving Hand Function and Functional Independence in Children with Unilateral Cerebral Palsy: A Feasibility Study. Cureus.

[35] Ziab H., Saleh S., Talebian S., Olyaei G., Mazbouh R., Sarraj A.R., Hadian M.R. (2024). Effectiveness of Virtual Reality Training Compared to Balance-Specific Training and Conventional Training on Balance and Gross Motor Functions of Children with Cerebral Palsy: A Double-Blinded Randomized Controlled Trial. J. Pediatr. Rehabil. Med..

[36] Meneses Castaño C., Penagos P., Jaramillo B.Y. (2023). Effectiveness of Robotic Technology and Virtual Reality for the Rehabilitation of Motor Function in Cerebral Palsy: Systematic Review. Rehabilitación.

[37] Komariah M., Hidayat R., Maulana S., Setiawan D., Mustika R., Nugraha B. (2024). Effectivity of Virtual Reality to Improve Balance, Motor Function, Activities of Daily Living, and Upper Limb Function in Children with Cerebral Palsy: A Systematic Review and Meta-Analysis. Children.

[38] Velasco Aguado J., Espada M.C., Muñoz-Jiménez J., Ferreira C.C., Gámez-Calvo L. (2025). Physical Exercise Interventions Using Virtual Reality in Children and Adolescents with Cerebral Palsy: Systematic Review. Healthcare.

[39] da Silva Ribeiro D.C., Silva R., Lima V., de Sousa Neto B.M., Lindquist A.R.R. (2021). Effectiveness of Virtual Reality Interventions of the Upper Limb in Children and Young Adults with Cerebral Palsy: A Systematic Review with Meta-Analysis. Braz. J. Phys. Ther..

[40] Warnier N., Lambregts S., Van de Port I. (2020). Effect of Virtual Reality Therapy on Balance and Walking in Children with Cerebral Palsy: A Systematic Review. Dev. Neurorehabilit..

[41] Cano de la Cuerda R. (2018). Nuevas Tecnologías en Neurorrehabilitación.

[42] Amirthalingam J., Paidi G., Alshowaikh K., Jayarathna I., Salibindla D.B.A.M.R., Karpinska-Leydier K., Ergin H.E. (2021). Virtual Reality Intervention to Help Improve Motor Function in Patients Undergoing Rehabilitation for Cerebral Palsy, Parkinson’s Disease, or Stroke: A Systematic Review of Randomized Controlled Trials. Cureus.

[43] Alrashidi M., Wadey C.A., Tomlinson R.J., Buckingham G., Williams C.A. (2023). The Efficacy of Virtual Reality Interventions Compared with Conventional Physiotherapy in Improving the Upper Limb Motor Function of Children with Cerebral Palsy: A Systematic Review of Randomised Controlled Trials. Disabil. Rehabil..

[44] Shoukat F., Ur Rehman S.S., Ahmed A. (2024). Effects of Physical Exercise Intervention on Improving Physical Functioning and Quality of Life among the Geriatric Population: A Systematic Review of Randomized Controlled Trials. J. Pak. Med. Assoc..

[45] Qian J., McDonough D.J., Gao Z. (2020). The Effectiveness of Virtual Reality Exercise on Individuals’ Physiological, Psychological and Rehabilitative Outcomes: A Systematic Review. Int. J. Environ. Res. Public Health.

[46] Yun H., Park M., Lee H., Choi E.K. (2024). Healthcare Interventions for Children Using Nonimmersive Virtual Reality: A Mixed Methods Systematic Review. J. Pediatr. Health Care.

[47] Cuschieri S. (2019). The CONSORT Statement. Saudi J. Anaesth..

[48] Hoffmann T.C., Glasziou P.P., Boutron I., Milne R., Perera R., Moher D., Altman D.G., Barbour V., Macdonald H., Johnston M. (2014). Better Reporting of Interventions: Template for Intervention Description and Replication (TIDieR) Checklist and Guide. BMJ.

